# Fetal Urinoma Secondary to Posterior Urethral Valve and Its Association to Postnatal Renal Function: A Multicenter Retrospective Study

**DOI:** 10.1002/pd.6773

**Published:** 2025-03-13

**Authors:** Flore‐Anne Martin, Matthieu Peycelon, Nicolas Vinit, Thibault Planchamp, Olivier Abbo, Maela Le Lous, Gwenaelle Le Bouar, Yves Ville, Annabelle Paye‐Jaouen, Thomas Blanc, Alexis P. Arnaud

**Affiliations:** ^1^ Department of Gynecology, Obstetrics and Reproductive Medicine CHU Rennes Université de Rennes Rennes France; ^2^ Department of Pediatric Urology National Reference Center for Rare Urinary Tract Malformations (MARVU) University Hospital Robert‐Debre APHP Université Paris Cité Paris France; ^3^ Department of Pediatric Surgery and Urology Hôpital Necker‐Enfants Malades APHP Université Paris Cité Paris France; ^4^ Department of Pediatric Surgery CHU Purpan Université de Toulouse Toulouse France; ^5^ Department of Obstetrics and Fetal Medicine Surgery and Imaging Hôpital Necker Enfants Malades AP‐HP Université Paris Cité Paris France; ^6^ Department of Pediatric Surgery CHU Rennes Université de Rennes Rennes France

## Abstract

**Aim:**

The role of prenatal urinoma in lower urinary tract obstruction (LUTO) such as posterior urethral valves (PUV) is debated. We aimed to describe the risk factors associated with fetal urinoma and the association between fetal urinoma and postnatal renal function before 2 years of age.

**Methods:**

This retrospective multicenter case‐control study from 2000 to 2018 included pregnant patients with suspected LUTO in their male fetus on prenatal ultrasound and postnatal confirmation of PUV. The exposure criterion was prenatal urinoma. The main composit outcome (MCO) was chronic kidney disease stage 3 or higher (CKD3+) before 2 years or death. Descriptive analyses of patient data and crude and multivariate logistic regression analyses were performed in an intent‐to‐treat fashion, thus including lost‐to‐follow‐up patients. Ethical approval # 20.144.

**Results:**

We included 299 patients, of whom 39 (13%) had prenatal urinoma. Thirty‐eight patients had a termination of pregnancy (12.7%). Sixty‐four (24.5%) patients'children were MCO positive. Twenty‐one children were lost‐to‐follow‐up, including one prenatal urinoma. Thirty‐nine (60.9%) of the remaining children had CKD3+ before the age of two, of whom 6 had a prenatal urinoma (9.4%). Among the 197 children negative to the MCO, 24 had a prenatal urinoma (12.2%, *p* = 0.42). Four died neonatally. In livebirth patients, prenatal urinoma was associated with obstetrical complications (*p* = 0.02), prenatal bloodcord sample for fetal beta2‐microglobulin (*p* = 0.01) and uro‐amniotic shunt (*p* = 0.01). Patients with prenatal urinoma more often presented with oligohydramnios (*p* = 0.01) and dilated posterior urethra (*p* = 0.01) and were less likely to have urinary tract infections (*p* = 0.02), although their DMSA scan was more often altered (*p* = 0.001). Prenatal urinoma was not significantly associated with CKD3+ before 2 years (OR = 0.56, CI98% = 0.20–1.39, *p* = 0.23).

**Conclusion:**

Renal function in infants with PUV was not worsened by the presence of a prenatal urinoma. Thus, there should not be any more pejorative message conveyed to concerned couples apart from other already known prenatal poor prognosis risk factors.

**Summary**

- The literature on prenatal urinoma in posterior urethral valves (PUV) is heterogeneous and inconsistent concerning its effect on renal function, with only a few comparative studies.
- With this large retrospective multicenter case‐control study, we provide more evidence‐based results on this topic to determine the effect of a prenatal urinoma in children/fetuses with PUV on postnatal renal function, to better counsel expecting couples on their future child's health, and to organize pregnancy and neonatal management.

## 1 Introduction

Urinoma, defined as a urine collection or leakage due to the rupture of the urinary tract [1, 2], can be diagnosed on prenatal ultrasound as an additional anechogenic intra‐abdominal structure around the kidney or next to the ureter or bladder. The main cause of urinoma in fetuses and newborns is lower urinary tract obstruction (LUTO) [3].

Posterior urethral valves (PUV) are the most common cause of LUTO in male fetuses and are found in 1/5000 to 1/8000 live births [4]. Their embryology may rely on incomplete regression of the urogenital membrane inside the posterior urethra, leading to obstruction of urinary flow, higher pressure in the urinary tract, and kidney hypodysplasia in severe cases [5]. PUV are suspected on prenatal ultrasound when bilateral hydronephrosis, dilated ureters, and a thickened and/or distended bladder with a dilated proximal urethra (“keyhole sign”) are observed in a male fetus [6]. The rate of chronic kidney disease (CKD) is up to 32% in patients with PUV [7], with a higher risk of CKD in cases of early gestational age at diagnosis [5]. Obstetricians may know better about renal function prenatally by performing a cord blood sample for beta2 microglobulin rate, that when elevated above 5 μg/L, is a sign of renal failure [8]. These investigations can help in planning management at birth as these neonates will need surgical endoscopic treatment as soon as possible with urinary diversion when needed. Otherwise, when a congenital disease with a particular severity and no cure (as is permanent bilateral renal damage) is diagnosed, expecting couples in France can ask for a termination of pregnancy (TOP) for medical reasons.

A “pop‐off” effect of the urinoma, supposedly protecting the kidney function in PUV, has been described in recent literature [4, 9, 10, 11]. It involves pressure relief inside the urinary cavities due to wall rupture. This theory was contested in studies identifying urinoma as a sign of dysplastic kidney, and therefore predictive of a higher morbi‐mortality [1, 12, 13], and in another paper concluding towards a null effect [14]. Both theories can result in very different prenatal and neonatal care strategies. The decision to terminate a pregnancy requires maximal certainty of prognosis to counsel couples and allow them to make the best decision for their family.

A recent review and meta‐analysis concluded that pop‐off mechanisms, in general, have a protective association towards renal function in PUV [15]. There is a need for specific evidence‐based results concerning the effect of prenatal PUV‐related urinoma on postnatal kidney function.

The primary objective of this study was to describe the association between renal function up to 2 years of age and prenatal ultrasound diagnosis of a urinoma following fetal suspicion of PUV. The maternal, fetal, and neonatal characteristics associated with this diagnosis are described as secondary objectives.

## 2 Methods

We retrospectively included patients whose child or fetus had a confirmed diagnosis of PUV between 2000 and 2018 with prenatal suspicion from four teaching tertiary centers in France: Robert‐Debré (Paris), Necker Enfants Malades (Paris), Rennes and Toulouse. The diagnosis of PUV had to be ascertained by postnatal cystography or by pathological examination performed by a board‐certified pathologist in TOP cases. Exclusion criteria included patients with other causes of LUTO and those whose prenatal data were unavailable. The ethics committee of our University Hospital approved the study protocol (#20.144). Non‐objection to data collection was required from all patients.

Prenatal ultrasound (performed by routine or reference practitioners) and pregnancy features were collected from medical reports. We classified gestational age at ultrasound finding or prenatal intervention as follows: “first trimester” before 14 gestational weeks (GW), “second trimester” between 14 and 28 GW, and “third trimester” after 28 GW. Premature birth was defined as a birth before 37 GW.

Pediatric follow‐up consisted of physical examinations and imaging, if necessary. Urinary tract infection (UTI) was defined as a fever ≥ 38.5°C with a positive urine culture for bacteria. Bladder dysfunction was suspected when several UTIs occurred with a significant post‐voiding residual volume on bladder ultrasound scan. Alteration of renal function on the DMSA scan was defined as a relative function difference of ≥ 10%, presence of renal scars, or decrease of ≥ 10% of absolute function in case of bilateral impairement. Biological data, such as serum creatinine levels, were collected every 3–6 months. Pediatric surgical data (imaging features, surgical approach, and cystoscopic observations) and fetal pathology data were also collected.

Our exposure variable was the occurrence of a prenatal urinoma, defined on ultrasound as a para‐urinary anechoic collection, intra‐abdominal effusion, or ascites that was not described as a renal cyst.

The main composit outcome (MCO) was occurrence of a postnatal chronic kidney disease stage 3 or more (CKD3+) for at least 3 months before age two, or occurrence of postnatal death. CKD3+ was defined as a glomerular filtration rate of 59 mL/min/1.73 m^2^ or less using the Schwartz formula.

Lost‐to‐follow‐up patients (*N* = 21, 8%) were analyzed as if they had the worst outcome; therefore, they were considered MCO patients in an intention‐to‐treat fashion.

Descriptive quantitative continuous variables are expressed as mean ± Standard Deviation [SD]). Descriptive quantitative discontinuous variables are expressed as medians (± range [Q1‐Q3]). Descriptive qualitative variables are presented as frequencies and percentages. We compared our patients' characteristics using the Chi‐square or Fisher tests when appropriate for categorical variables and t‐tests for quantitative continuous variables.

We performed crude and adjusted logistic regression models to study the association between covariables, exposure, and outcome. These analyses were performed on our live‐birth population. Patients with missing data for specifically selected variables were excluded from the analysis. Crude and adjusted logistic regression models were also performed using exclusion of the lost‐to‐follow‐up patients in order to rule out any bias coming from their inclusion. The adjustment strategy for potential confounders was established using a Directed Acyclic Graph [16] based on the variables associated with both the outcome and exposure criteria (*p* < 0.10) in the crude analyses. The selected adjustment variables were: the “key‐hole sign” on a prenatal ultrasound (yes or no), prenatal ultrasound signs of kidney dysplasia (yes or no) and performance of a cord blood sample for fetal beta2 microglobulin (yes or no). The effects were expressed as odds ratios (OR) with 95% confidence intervals (CI). OR was considered statistically significant when the CI did not include 1. Data were managed in an Excel database (Microsoft, Redmond, WA, USA) and analyzed using the free software R (version 3.5.0).

## 3 Results

During the study period, 299 patients expecting a fetus with prenatal signs of LUTO and postnatal confirmation of a PUV were reviewed. Prenatal urinoma was observed in 39 patients (13%). Thirty‐eight pregnancies (12.7%) were terminated, including 7 with prenatal urinomas. None of the prenatal characteristics collected in our study was significantly associated with the occurrence of urinoma in our TOP population. No intrauterine fetal death was observed. Four neonates died within the first week of life, including one with prenatal urinoma.

**FIGURE 1:**
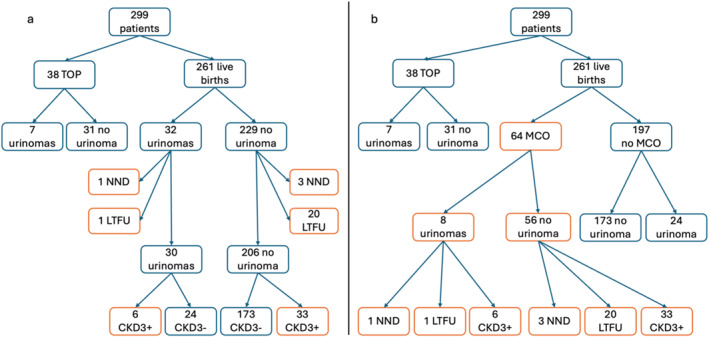
Flowcharts of the population. (a) flowchart depending on the presence of a urinoma. (b) flowchart depending on the main composit outcome. LTFU, lost to follow‐up; MCO, main composit outcome; NND, neonatal death; TOP, termination of pregnancy. Boxes in orange correspond to the MCO.

Sixty‐four (24.5%) children of patients were in the MCO group. Twenty‐one (32.8%) children of patients were lost to follow‐up before two years with a median age at last follow‐up of 12 months [9–17]. Among them, 1 patient had prenatal urinoma. Thirty‐nine (60.9%) of the remaining children had CKD3+ before the age of two, of whom 6 had a prenatal urinoma (9.4%). Among the 197 children negative to the MCO, 24 had a prenatal urinoma (12.2%, *p* = 0.42). cf Figure 1a and b.

### 3.1 Prenatal Characteristics

Prenatal urinoma was diagnosed during the second trimester in 44% of the cases (*n* = 16) and during the third trimester in 56% (*n* = 20) (3 had unavailable diagnosis timing). Seven pregnancies (17.9%) were terminated after a prenatal urinoma diagnosis. A “key‐hole” sign was more often visualized in patients diagnosed with prenatal urinoma: 63.2% versus 40.7% (*p* = 0.01). There were more amniotic fluid abnormalities associated with prenatal urinoma: 17 of 39 (43.6%) had an oligohydramnios versus 53 of 260 (20.6%) (*p* < 0.01). Fetuses with a prenatal urinoma underwent vesicoamniotic shunting more often (25.6% vs. 3.5%, *p* < 0.01) as well as cord blood sampling for fetal beta2 microglobulin level (59% vs. 25.8%, *p* < 0.01).

The prenatal keyhole sign was more often observed in children of the MCO group (56.7% vs. 39%, *p* = 0.02), especially if seen early in pregnancy (= 0.048), as well as bilateral renal dysplasia on prenatal ultrasound (21.9% vs. 9.8%, *p* = 0.048). Patients' fetuses were in the MCO group were more likely to get a cord blood sample for fetal serum beta2 microglobulin (41.9% vs. 23.4%, *p* < 0.01) with a higher (≥ 5 μg/L) initial fetal serum level (52.9% vs. 20.5%, *p* = 0.015). Further data on prenatal features are presented in Tables 1, 2, 3 to 4.

**TABLE 1:** Descriptive analysis and crude logistic regression of prenatal ultrasound features' association with chronic kidney disease stage 3 or more up to 2 years of age in our live birth population. Fisher or Chi‐square tests were used to test the associations with qualitative variables.

		No CKD3+ (*N* = 197)		CKD3+ before 2 years old (*N* = 64)		Descriptive analysis (Fisher or Chi^2^ test)	Univariate logistic regression	
		*N*	%	*N*	%	*p*	OR	*p*
Prenatal urinoma	None	173	87.8	56	87.5	0.42	Ref	Ref
	Any	24	12.2	8	12.5	1	1.03	0.95
	Unilateral	9	4.6	6	9.4		2.07	0.19
	Bilateral	3	1.5	0	0.0		0.00	0.97
	Ascitis	12	6.1	2	3.1		0.06	0.47
Prenatal hydronephrosis	None	8	4.1	1	1.6	0.64	Ref	Ref
	Unilateral	20	10.3	5	7.8		2.00	0.55
	Bilateral	167	85.6	58	90.6		2.78	0.34
	NA	2	—	0	—			
Gestational age at prenatal hydronephrosis finding	First trimester	2	1.1	0	0.0	< 0.01	Ref	Ref
	Second trimester	56	31.1	32	54.2		0.00	0.99
	Third trimester	122	67.8	27	45.8		0.00	0.99
	NA	7	—	4	—			
Prenatal renal dysplasia	None	162	83.9	44	68.8	0.03	Ref	Ref
	Unilateral	12	6.2	6	9.4		1.84	0.25
	Bilateral	19	9.8	14	21.9		2.71	0.01
	NA	4	—	0	—			
Prenatal ureteral enlargement	None	62	32.6	16	25.0	0.51	Ref	Ref
	Unilateral	17	8.9	6	9.4		1.37	0.57
	Bilateral	111	58.4	42	65.6		1.47	0.25
	NA	7	—	0	—			
Prenatal bladder size	Normal	88	46.3	22	36.7	0.41	Ref	Ref
	Small	9	4.7	3	5.0		1.33	0.68
	Large	93	48.9	35	58.3		1.51	0.19
	NA	7	—	4	—			
Prenatal bladder thickening	Normal	89	48.1	29	49.2	0.89	Ref	Ref
	Thick	96	51.9	30	50.8		0.96	0.89
	NA	12	—	5	—			
Gestational age at prenatal bladder abnormality finding	First trimester	2	1.6	0	0.0	< 0.01	Ref	Ref
	Second trimester	31	24.8	24	54.5		0.00	0.99
	Third trimester	92	73.6	20	45.5		0.00	0.99
	NA	10	—	0	—			
Prenatal dilated prostatic urethra	No	114	61.0	26	43.3	0.02	Ref	Ref
	Yes	73	39.0	34	56.7		2.04	0.02
	NA	10	—	4	—			
Gestational age at prenatal dilated urethra finding	Second trimester	20	28.6	16	48.5	0.05	Ref	Ref
	Third trimester	50	71.4	17	51.5		0.43	0.05
	NA	13	—	5	—			
Amniotic fluid quantity	Normal	129	66.2	31	49.2	0.06	Ref	Ref
	Oligoanamnios	39	20.0	22	34.9		2.35	0.01
	Anamnios	18	9.2	8	12.7		1.85	0.19
	Hydramnios	9	4.6	2	3.2		0.92	0.92
	NA	2	—	1	—			

**TABLE 2:** Descriptive analysis and crude logistic regression association of pregnancy characteristics in our population (livebirth and TOP) and prenatal urinoma findings. Fisher or Chi square tests were used to test the association with qualitative variables.

		Population (*N* = 299)		No prenatal urinoma (*N* = 260)		Prenatal urinoma (*N* = 39)		Descriptive analysis (Fisher or Chi^2^ test)	Univariate logistic regression	
		*N*	%	*N*	%	*N*	%	*p*	OR	*p*
Prenatal hydronephrosis	None	24	8.1	18	6.9	6	15.8	0.072	Ref	Ref
	Unilateral	26	8.7	21	8.1	5	13.1		0.71	0.23
	Bilateral	247	83.2	220	84.9	27	71.1		0.37	0.05
	NA	2	—	1	—	1	—			
Prenatal renal dysplasia	None	222	75.3	199	77.4	23	60.5	0.056	Ref	Ref
	Unilateral	18	6.1	15	5.8	3	7.9		1.73	0.41
	Bilateral	55	18.6	43	16.7	12	31.6		2.41	0.03
	NA	4	—	3	—	1	—			
Prenatal ureteral enlargement	None	98	33.6	81	31.9	17	44.7	0.308	Ref	Ref
	Unilateral	24	8.3	22	8 0.6	2	5.3		0.43	0.29
	Bilateral	170	58.2	151	59.4	19	50.0		0.60	0.16
	NA	7	—	6	—	1	—			
Prenatal bladder size	Normal	111	38.4	101	40.4	10	25.6	0.2	Ref	Ref
	Small	12	4.2	10	4.0	2	5.1		2.02	0.40
	Large	165	57.1	139	55.6	26	66.7		1.89	0.11
	NA	11	—	10	—	1	—			
Prenatal bladder thickening	Normal	142	50.9	127	52.9	15	38.5	0.094	Ref	Ref
	Thick	137	49.1	113	47.1	24	61.5		1.80	0.10
	NA	20	—	20	—	0	—			
Prenatal dilated prostatic urethra	No	160	56.3	146	59.3	14	36.8	0.009	Ref	Ref
	Yes	124	43.7	100	40.7	24	63.2		2.50	0.01
	NA	15	—	14	—	1	—			
Amniotic fluid quantity	Normal	175	59.1	166	64.6	9	23.1	< 0.0001	Ref	Ref
	Oligoanamnios	70	23.6	53	20.6	17	43.6		5.92	< 0.01
	Anamnios	40	13.5	30	11.7	10	25.6		6.15	< 0.01
	Hydramnios	11	3.7	8	3.1	3	7.7		6.92	0.01
	NA	3	—	3	—	0	—			

**TABLE 3:** Descriptive analysis and crude logistic regression of prenatal interventions' association with chronic kidney disease stage 3 or more up to 2 years of age in our live birth population. Fisher or Chi square tests were used to test the association with qualitative variables.

		No CKD3+ (*N* = 197)		CKD3+ before 2 years old (*N* = 64)		Descriptive analysis (Fisher or Chi^2^ test)	Univariate logistic regression	
		*N*	%	*N*	%	*p*	OR	*p*
Fetal uro‐amniotic shunt	No	184	94.8	59	93.7	0.75	Ref	Ref
	Yes	10	5.2	4	6.3		1.25	0.72
	NA	3	—	1	—			
Fetal Beta2globulin cordblood sample	No	147	76.6	36	58.1	< 0.01	Ref	Ref
	Yes	45	23.4	26	41.9		2.36	0.01
	NA	5	—	2	—			
First fetal beta2globulin level	< 5	31	79.5	8	47.1	0.02	Ref	Ref
	≥ 5	8	20.5	9	52.9		4.36	0.02
	NA	158	—	47	—			
Gestational age at first fetal beta2globulin sampling	Second trimester	7	17.1	8	38.1	0.07	Ref	Ref
	Third trimester	34	82.9	13	61.9		0.33	0.07
	NA	4	—	3	—			

**TABLE 4:** Descriptive analysis and crude logistic regression association of prenatal interventions in our population (livebirth and TOP) and prenatal urinoma findings. Fisher or Chi square tests were used to test the association with qualitative variables.

		Population (*N* = 299)		No prenatal urinoma (*N* = 260)		Prenatal urinoma (*N* = 39)		Descriptive analysis (Fisher or Chi^2^ test)	Univariate logistic regression	
		*N*	%	*N*	%	*N*	%	*p*	OR	*p*
Fetal uro‐amniotic shunt	No	276	93.6	247	96.5	29	74.4	< 0.001	Ref	Ref
	Yes	19	6.4	9	3.5	10	25.6		9.46	0.00
	NA	4	—	4	—	0	—			
Fetal Beta2globulin cord blood sample	No	203	69.8	187	74.2	16	41.0	< 0.001	Ref	Ref
	Yes	88	30.2	65	25.8	23	59.0		4.14	0.00
	NA	8	—	8	—	0	—			
First fetal beta2globulin rate (μmol/L)	< 5	40	56.3	25	50.0	15	71.4	0.1	Ref	Ref
	≥ 5	31	43.7	25	50.0	6	28.6		0.40	0.10
	NA	228	—	210	—	18	—			

### 3.2 Neonatal Characteristics

There were fewer premature births when a prenatal urinoma was found (42.1% vs. 66.3%, *p* = 0.004). Patients' babies with prenatal urinoma had a significantly higher rate of neonatal intensive care stay (53.1% vs. 22.3%, *p* = 0.007), as did those who were in the MCO group (45.8% vs. 24%, *p* = 0.007). There was no other significant difference in terms of birth and neonatal characteristics between the two groups. The mean creatinine nadir during the neonates' first month was 0.75 mg/dL (±0.5), with a mean of 0.64 (±0.3) in the urinoma group versus 0.76 (±0.5) in the group without prenatal urinoma (*p* = 0.1) (Table 5).

**TABLE 5:** Descriptive analysis and crude logistic regression of neonatal characteristics, postnatal imagery features, first surgery characteristics as well as pediatric medical characteristics' association with prenatal urinoma findings in our population (livebirth and TOP). Fisher’s exact or Chi squared tests were used to test the association with qualitative variables and student's t‐test for quantitative variables.

		Livebirth population (*N* = 261)		No prenatal urinoma (*N* = 229)		Prenatal urinoma (*N* = 32)		Descriptive analysis (Fisher or Chi^2^ test or Student's t test)	Univariate logistic regression	
		*N*	%	*N*	%	*N*	%	*p*	OR	*p*
Intensive care at birth	No	161	73.2	146	77.7	15	46.9	< 0.001	Ref	Ref
	Yes	59	26.8	42	22.3	17	53.1		3.94	< 0.001
	NA	79	—	72	—	7	—			
Hydronephrosis at birth	None	13	5.6	8	3.9	5	18.5	< 0.001	Ref	Ref
	Unilateral	21	9	16	7.7	5	18.5		0.5	0.366
	Bilateral	199	85.4	182	88.3	17	63.0		0.15	< 0.001
	NA	28	—	23	—	5	—			
Renal dysplasia at birth	None	147	64.2	129	63.9	18	66.7	0.15	Ref	Ref
	Right	30	13.1	24	11.8	6	22.2		1.79	0.263
	Bilateral	52	22.7	49	24.3	3	11.1		0.44	0.20
	NA	32	—	27	—	5	—			
Urinoma at birth	None	196	87.1	190	94.1	6	26.1	< 0.001	Ref	Ref
	Unilateral	19	8.4	9	4.5	10	43.5		35.18	< 0.001
	Bilateral	4	1.8	2	1.0	2	8.7		31.67	0.001
	Ascitis	6	2.7	1	0.5	5	21.7		158.33	< 0.001
	NA	36	—	27	—	9	—			
Bladder size at birth	Normal	45	23.4	38	22.9	7	26.9	0.8	Ref	Ref
	Small	101	52.6	87	52.4	14	53.8		0.87	0.79
	Large	46	24.0	41	24.7	5	19.2		0.66	0.51
	NA	69	—	63	—	6	—			
Vesicoureteral reflux	No	97	50.3	80	51.3	17	46	0.87	Ref	Ref
	Unilateral	76	39.4	62	39.7	14	37.8		0.82	0.669
	Bilateral	20	10.3	14	9	6	16.2		0.61	0.531
	NA	68	—	10	—	20	—			
Cystoscopic bladder size	Normal	45	47.9	39	47.6	6	50.0	0.61	Ref	Ref
	Small	30	31.9	25	30.5	5	41.7		1.30	0.69
	Large	19	20.2	18	22.0	1	8.3		0.36	0.36
	NA	167	—	147	—	20	—			
Surgical treatment of valves	None	5	2.6	5	2.9	0	0.0	0.9	Ref	Ref
	Blade section	125	63.8	109	63.0	16	69.6		0.00	0.99
	Electric resection	66	33.7	59	34.1	7	30.4		0.00	0.99
	NA	65	—	56	—	9	—			
Posthectomy	No	179	72.8	159	73.6	20	66.7	0.42	Ref	Ref
	Yes	67	27.2	57	26.4	10	33.3		1.39	0.43
	NA	15	—	13	—	2	—			
Urinary infection before age 2	No	70	29.5	56	26.9	14	48.3	0.02	Ref	Ref
	Yes	167	70.5	152	73.1	15	51.7		0.39	0.02
	NA	24	—	21	—	3	—			
Chronic renal disease stage 3	No	197	75.5	173	75.5	24	75	0.95	Ref	Ref
	Yes	64	24.5	56	24.5	8	25		1.03	0.95
Creatinine levels age 1 (μmol/mL)		59.4	61.4	60	62.9	54.3	50.9	0.78	0.99	0.49
Age at chronic renal disease stage 3	First month	27	42.9	23	41.8	4	50	0.51	Ref	Ref
	< 12 months old	19	30.2	18	32.7	1	12.5		0.32	0.36
	12–24 months old	17	26.9	14	25.5	3	37.5		1.23	0.80
	NA	2	—	2	—	0	—			
DMSA scan	Normal	31	29.2	28	31.5	3	17.6	0.03	Ref	Ref
	Unilateral impairment	70	66	59	66.3	11	64.8		1.74	0.42
	Bilateral impairment	5	4.7	2	2.2	3	17.6		14	0.02
	NA	155	—	140	—	15	—			

### 3.3 Pediatric Imaging, Surgery and Follow‐Up

Most prenatal results on urinomas were consistent after birth: 96% of patients' children with no prenatal diagnosis of urinoma did not have any on the first postnatal ultrasound scan, whereas 74% of patients' children who did have a postnatal confirmation. The presence of a prenatal urinoma was associated with an increased risk of bilateral renal impairment on postnatal DMSA scan (17.6% vs. 2.2%, *p* < 0.001) and a lower risk of UTI before 2 years of age (51.7% vs. 73.1%, *p* = 0.02) (see Table 5). Postnatal reflux was not associated with a prenatal diagnosis of urinoma, nor was its stage or laterality compared with urinoma.

Signs of bilateral renal dysplasia on the first postnatal ultrasound were more often observed in patients' children of the MCO group (35.8% vs. 18.8%, *p* = 0.03).

Neonates who did not undergo successful valve ablation during their first cystoscopy had an increased risk of CKD3+ (11.4% vs. 0%, *p* = 0.001). These 5 patients underwent a urinary diversion and a successful secondary endoscopic valve resection.

CKD3+ before the age of two was associated with less thriving as shown by the significantly lower height at two (80.5 cm (+/− 14) versus 87.6 cm (+/− 4), *p* < 0.001), UTI before age two (84% vs. 66.8%, *p* = 0.02) and bladder dysfunction (35.7% vs. 9.5%, *p* < 0.001) (see Table 6).

**TABLE 6:** Descriptive analysis and crude logistic regression of neonatal characteristics. Postnatal imagery features. First surgery characteristics as well as pediatric medical characteristics' association with and chronic kidney disease stage 3 or more up to 2 years of age in our livebirth population. Fisher’s exact or Chi squared tests were used to test the association with qualitative variables and student's t test for quantitative variables.

		No CKD3+ (*N* = 197)		CKD3+ before 2 years old (*N* = 64)		Descriptive analysis (Fisher or Chi^2^ test)	Univariate logistic regression	
		*N* or mean	% or SD	*N* or mean	% or SD	*p*	OR	*p*
Intensive care at birth	No	117	76.0	26	54.2	< 0.01	Ref	Ref
	Yes	37	24.0	22	45.8		2.68	< 0.01
	NA	43	21.8	16	25.0	0.72		
Hydronephrosis at birth	None	10	5.6	3	5.6	0.5	Ref	Ref
	Unilateral	14	7.8	7	13.0		1.67	0.53
	Bilateral	155	86.6	44	81.5		0.95	0.94
	NA	18	—	10	—			
Renal dysplasia at birth	None	119	67.6	28	52.8	0.03	Ref	Ref
	Unilateral	24	13.6	6	11.3		1.06	0.90
	Bilateral	33	18.8	19	35.8	0.01	2.45	0.01
	NA	21	—	11	—			
Urinoma at birth	None	151	87.3	45	86.5	0.22	Ref	Ref
	Unilateral	12	6.9	7	13.5		1.96	0.18
	Bilateral	4	2.3	0	0.0		0.00	0.99
	Ascitis	6	3.5	0	0.0		0.00	0.99
	NA	24	—	12	—			
Vesicoureteral reflux	No	80	51.3	17	45.9	0.448	Ref	Ref
	Unilateral	62	39.7	14	37.8		1.06	0.88
	Bilateral	14	9.0	6	16.2		2.02	0.21
	NA	41	—	27	—			
Cystoscopic bladder size	Normal	36	23.8	9	22.0	0.1	Ref	Ref
	Small	78	51.7	23	56.1		3.73	0.05
	Large	37	24.5	9	22.0		2.73	0.19
	NA	46	—	23	—			
Primary surgical treatment of valves	None	0	0.0	5	11.4	< 0.01	Ref	Ref
	Blade section	101	66.4	24	54.5		0.00	0.99
	Electric resection	51	33.6	15	34.1		0.00	0.99
	NA	45	—	20	—			
Posthectomy	No	142	75.5	37	63.8	0.08	Ref	Ref
	Yes	46	24.5	21	36.2		1.75	0.08
	NA	9	—	6	—			
Height at 2 years old (cm)		87.6	4.3	80.5	14.2	< 0.01	0.790	< 0.01
	NA	97.0	—	39.0	—			
Urinary infection before age 2	No	62	33.2	8	16.0	0.02	Ref	Ref
	Yes	125	66.8	42	84.0		2.60	0.02
	NA	10	—	14	—			
Creatinine levels age 1 (μmol/mL)		38.6	15.4	92.7	88.1	< 0.01		
Bladder dysfunction	No	152	90.5	18	64.3	< 0.01	Ref	Ref
	Yes	16	9.5	10	35.7		5.28	< 0.01
	NA	29	—	36	—			
Bladder dysfunction treatment	None	162	97.6	22	78.6	< 0.01	Ref	Ref
	Repetitive catheterization	2	1.2	3	10.7		11.05	0.01
	Incontinent cystostomy	2	1.2	3	10.7		11.05	0.01
	NA	3	—	1	—			

There was no other significant difference in the surgical procedures, clinical follow‐up, or postnatal imaging features.

### 3.4 Logistic Regression Analyses

Crude logistic regression analyses showed a protective association between the occurrence of prenatal urinoma and a maternal age above 35 years (OR = 0.39, CI 95% = 0.14–0.91, *p* = 0.04), postnatal bilateral hydronephrosis (OR = 0.15, CI = 0.04–0.54, *p* < 0.001), premature birth (OR = 0.37, CI = 0.18–0.74, *p* = 0.01), and UTI before age two (OR = 0.39, CI = 0.18–0.88, *p* = 0.02).

Occurrence of prenatal urinoma was associated with bilateral signs of renal dysplasia on prenatal ultrasound (OR = 2.41, CI = 1.09–5.16, *p* = 0.03), amniotic fluid abnormalities (among which oligohydramnios OR = 5.92, CI = 2.54–14.63, *p* < 0.001) and a prenatal “key‐hole” sign (OR = 2.5, CI = 1.25–5.19, *p* = 0.01). These patients were also more concerned with a prenatal cord blood sample (OR = 4.14, CI = 2.07–8.44, *p* < 0.001), a prenatal uro‐amniotic shunt procedure (OR = 9.46, CI = 3.54–25.74, *p* < 0.001), need for intensive care at birth (OR = 3.94, CI = 1.82–8.64, *p* < 0.001), and bilateral renal impairment on DMSA scan (OR = 14, CI = 1.72–1484.99, *p* = 0.02).

Crude logistic regression analyses showed a significant association between the MCO and failure to thrive (OR = 0.79, CI = 0.69–0.88, *p* < 0.001). Bilateral signs of renal dysplasia on prenatal ultrasound (OR = 2.71, CI = 1.24–5.82, *p* = 0.01), a “key‐hole” sign (OR = 2.04, CI = 1.14–3.71, *p* = 0.02), a fetal beta2 microglobulin level ≥ 5 μg/L (OR = 4.36, CI = 1.3–15.53, *p* = 0.02) and bilateral signs of renal dysplasia on postnatal ultrasound (OR = 2.45, CI = 1.21–4.92, *p* = 0.01) were associated with a higher risk of CKD3+ before age two. UTI before age two (OR = 2.6, CI = 1.21–6.28, *p* = 0.02) and bladder dysfunction (OR = 5.28, CI = 2.05–13.36, *p* < 0.001) were associated with a higher risk of MCO. These associations remained significant when running the analyses without the lost‐to‐folluw‐up population, and no other significant association showed up.

In the multivariate logistic regression analysis, we showed that prenatal urinoma was not an independent risk factor for MCO in early infancy but tended towards a protective association (OR = 0.56, CI 98% = 0.20–1.39, *p* = 0.23). This result was similar in the model without the lost‐to‐follow‐up population (OR = 0.62, CI 95% = 0.2–1.7, *p* = 0.38).

## 4 Discussion

In our study, we observed that prenatal urinoma diagnosed in cases of prenatal suspicion of PUV did not significantly impact postnatal renal function up to 2 years old after adjusted analyses. It may be a protective factor against postnatal urinary infections and bilateral hydronephrosis in children born with PUV but is associated with a higher risk of intensive care at birth and bilateral renal alterations on DMSA scan.

The effect of prenatal urinoma on kidney function is a subject of debate. The negative effect reported by some authors [1, 10, 12, 13] may be explained by the difference in the main outcome criteria. The authors chose either renal function of the kidney homolateral to the urinoma, a DMSA scan, or prophylactic nephrectomy as the main outcome. Our goal in this study was to determine the message that should be conveyed to concerned expecting couples. In France, prenatal counseling may lead to TOP, which can only be accepted by a dedicated, regionally approved, multidisciplinary prenatal committee if the diagnosis meets a certain and specific severity [17]. This type of diagnosis concerns only bilateral impairment of renal function, which is why we chose this outcome. Other differences in the literature may be explained by the chosen definitions of urinoma. For instance, D'Oro et al. included ureteral reflux as a “pop‐off” sign and assigned it the same significance as a urinoma [18]. Lundar et al. used a different outcome than ours: normal renal function was classified as glomerular filtration rate (GFR) > 90 mL/min/1.73 m^2^ at the last follow‐up. In their results, nine (18.8%) patients had moderate CKD (grade 3), one (2.1%) had severe CKD (grade 4), and five patients (10.4%) had end‐stage CKD (grade 5), with 31.3% CKD 3+ versus 17.9% in our study. Moreover, they did not use the intention‐to‐treat principle with regard to the classification of lost‐to‐follow‐up patients, meaning that they might have underrated their outcomes [19]. Finally, Heikkila et al. reported a higher end‐stage CKD rate, which was present in four of their 16 patients (25%) with urinoma and in 16 of their 69 controls (23%). However, their results focused on the whole childhood [20]. One could think that following the choice of an intent‐to‐treat strategy, including the 21 lost‐to‐follow‐up patients with only 1 urinoma, could have overrated the main outcome in the worse direction. However, the analysis without this subgroup showed similar results, reinforcing our results and methods.

Some of our results are similar to those found in the recent literature. Holmes et al. concluded that prenatal uroamniotic shunt intervention had no statistically significant benefit on postnatal renal function [21]. Rianthavorn et al. noted that growth retardation could be observed in boys with PUV when CKD began early in life [22]. Berte et al. found a CKD rate of 20% at the age of 5 years, which approached our rate of 17.9% at the age two [23]. Ylinen et al. reported that 8 of 23 prenatally detected cases (35%) who developed poor renal function were classified as stage 4 CKD [24].

As for Baipai et al., our most common abnormal prenatal ultrasonographic finding was hydronephrosis (70.8% in their study and 98% in the present study), and we observed similar rates of vesicoureteral reflux (50.8% and 49.7%, respectively). However, our series differed in terms of renal dysplasia (55/299 (18.6%) versus 0 (0.0%)) and unilateral dysplasia (18/299 (6.1%) versus 11/65 (16.9%)) [25].

We found that a prenatal urinoma diagnosis was significantly associated with intensive care at birth. This may be explained by the fact that a urinoma can have a compressive impact on abdominal and thoracic organs. Prenatal puncture of a urinoma may be needed to avoid obstetric complications during labor and vital complications at birth [26]. Nevertheless, this type of puncture can only be temporarily beneficial if performed too early in pregnancy, with potential recollection of the urinoma [13]. Patil et al. had such results concerning neonatal distress with “abdominal distention in 55.5% of cases, flank mass in 55.5%, respiratory distress in 16.6% and septicemia in 22.2%”. Their explanation for this phenomenon was that extravasation could only be beneficial when extracapsular, whereas subcapsular renal extravasation may cause more damage. They also suggested a decompression strategy when confronted with a compressive urinoma, potentially with a shunt if there was a relapse [10]. Nevertheless, there were less preterm births in the cases of prenatal urinoma. This might be a sign of a decrease in pressure in urinary cavities and its consequence on the renal tissue, with less risk of oligoanhydramnios and renal dysplasia which are the main features in this situation that can lead to obstetrical decision for induced preterm birth.

Evolution and location of the urinoma may influence its impact. Massicot et al. and Yitta et al. suggested that the prenatal regression of a urinoma could be a sign of oligoanuria and poor renal clearance [9, 13]. However, according to Gorincour et al., it depends solely on the location of the urinoma. Ascites would be protective of renal function, whereas an encapsulated retroperitoneal urinoma would not relieve pressure in the urinary cavities [12]. However, we did not obtain similar results.

In our study, the occurrence of a urinoma was associated with signs of oligohydramnios and visualization of a ”key‐hole” sign on an ultrasound scan and the need for fetal cord blood beta2 microglobulin sample. Despite being associated with these poor prognosis factors, a prenatal urinoma did not seem to worsen postnatal renal prognosis, which supports the hypothesis of a potential “pop‐off” effect. This supposition is reinforced by our results showing a protective association towards postnatal hydronephrosis, suggesting that the pressure inside the urinary cavities is relieved. Going further with this protective association towards postnatal dilatation, it might also explain the protective association with UTI.

To date, our cohort is the largest to focus on prenatal urinoma in PUV, which is a major strength of our study. The multicenter collection of data conveyed a thorough recruitment of patients, which minimized selection bias. We add to the sparse literature on this topic, which requires additional original articles to confirm the hypotheses.

Despite our large sample size, our primary results did not reach statistical significance. This might be explained by the inherent recruitment bias in our study, as it was retrospective. Moreover, urinomas can be difficult to identify and confirm, and several differential diagnoses exist, including kidney cysts and intestinal duplication [9, 27]; therefore, there is a possible classification bias. We can also state a chronological bias, as our recruitment was from 2000 to 2018, a period during which ultrasound technologies and surgical techniques evolved. However, there was no significant association between the date of birth before or after 2005 (year of national guidelines for systematic ultrasound prenatal surveillance in France) and the occurrence of prenatal urinoma or MCO in PUV. Therefore, this bias is most likely nondifferential. A differential recruitment bias towards the null effect might exist, as none of the one center's patients were affected by TOP or were lost to follow‐up. Finally, two years old may be too early to assess kidney function, as in the medical literature we find 26%–30% of adolescents with advanced CKD after diagnosis of PUV (either pre‐ or postnatal) [28].

Our results can only apply to prenatal diagnosis, keeping in mind that clear diagnosis of PUV cannot be achieved during pregnancy at the moment and requires postnatal confirmation. However, one can come close to a diagnosis of PUV in the prenatal period using the Z‐score of fetal bladder size [29] although there is no specific ultrasound sign of PUV [30].

## 5 Conclusion

When a LUTO suspected to be a PUV on a prenatal ultrasound scan is complicated with urinoma, there should not be any more pejorative message conveyed to concerned couples apart from other already known prenatal poor prognosis risk factors. Our study's results add to the current literature on this very specific topic, which could be addressed by performing a meta‐analysis gathering the different populations published in the last few decades.

## Ethics Statement

The study protocol was approved by the Ethics Committee of our University Hospital (#20.144). Non‐objection to data collection was required from all patients.

## Conflicts of Interest

The authors declare no conflicts of interest.

## Data Availability

The data supporting the findings of this study are available from the corresponding author upon reasonable request.
